# Long-Term Formaldehyde Emission Potential from UF- and NAF-Bonded Particleboards

**DOI:** 10.3390/polym12081852

**Published:** 2020-08-18

**Authors:** Charles R. Frihart, Timothy L. Chaffee, James M. Wescott

**Affiliations:** 1 US Forest Products Laboratory, Madison, WI 53726, USA; 2 Solenis LLC, Wilmington, DE 19803, USA; tlchaffee@solenis.com; 3 Wescott Consulting, Minocqua, WI 54548, USA; wescottj@icloud.com

**Keywords:** soy, no-added formaldehyde, NAF, urea formaldehyde, UF, formaldehyde emissions, heat, humidity

## Abstract

As a result of the dominance of urea formaldehyde (UF)-bonded particleboard, it seemed worthwhile to examine formaldehyde emissions years after production. A California Air Resources Board (CARB) phase II-compliant commercial particleboard produced with a UF resin adhesive was compared to a no-added formaldehyde (NAF)-particleboard produced with Soyad™ adhesive resin for formaldehyde emissions during exposure to elevated humidity and temperature conditions after being in a room at 21 ± 1.9 °C, 50 ± 3.3% relative humidity for 3.5 years. A modified version of EN 717-3 was used to collect formaldehyde emissions under typical along with higher temperature and humidity conditions. The formaldehyde emissions from the commercial particleboard panel bonded with a UF adhesive even after the 3.5 years of exposure greatly increased only during exposure of the panels to elevated heat and humidity compared to typical testing conditions. The amounts were the same as those with the previous shorter-term study. In contrast, formaldehyde emissions from the NAF-bonded particleboard were not as susceptible (in absolute terms) to increases in temperature and relative humidity conditions.

## 1. Introduction

Due to adhesive costs, manufacturing robustness, and generally good bond performance, urea-formaldehyde (UF) resins are the dominant adhesive for manufacturing interior wood products, such as interior plywood, particleboard, and fiberboard [1]. Unlike other formaldehyde-based adhesives, the UF adhesive is not totally stable, especially at exposures to greater than the typical testing temperatures and humidity of 25 °C and 50% relative humidity [2]. 

**Figure 1:**
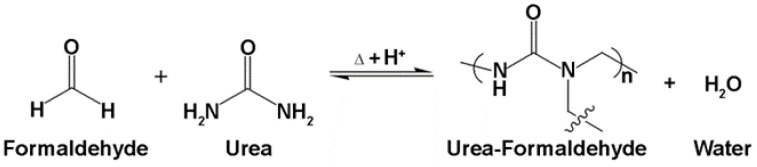
The reversible reaction of urea and formaldehyde for providing the urea–formaldehyde polymer that can be hydrolyzed to liberate formaldehyde.

Research has shown that the urea-formaldehyde condensation polymerization reaction that produces water has a significant reverse potential [3], as illustrated in Figure 1. The more water there is, the more the stoichiometry favors the reverse reaction; the presence of acid and heat accelerates this reaction. Thus, a UF adhesive has a significant likelihood of depolymerization and emission of formaldehyde unlike the other formaldehyde-containing wood adhesives. Further studies demonstrated that as the heat and humidity increased, the formaldehyde release increased, as would be expected by the reversible reaction chemistry subject to polymer bond breakage via hydrolysis [4,5]. Right after production, the board products can have high formaldehyde emissions, but these decay to a lower value. The decay curves vary depending on the production characteristics and are commonly described as exponential and more recently as power-law decay [6], but the different decay curves level out and plateau above zero. Thus, the emphasis on setting lower emission limits of the bonded products has continued, because the basic chemistry issue has not been solved. 

The original UF had a low urea-to-formaldehyde ratio of about 1:1.17 for the final product, which led to strong products and rapid cure conditions, but these products produced a detectable formaldehyde odor. Formaldehyde has been a health concern, especially when released into the home environment. The 1980s had considerable studies on cured UF adhesive stability [7] identifying wood composites as a source of indoor formaldehyde emissions [8] as well as work on formaldehyde testing methods [6,9,10,11,12,13,14,15,16]. There has been a long-term decline in the emissions from particleboard, especially as countries look at tightening the allowed emissions [17,18,19,20,21,22]. The emphasis was outside North America until formaldehyde was reclassified from a suspected to a known carcinogen [23,24]. Thus, by law, the CARB (California Air Resources Board) adopted standards intended to significantly reduce and regulate formaldehyde emissions in composite wood products [25]. The CARB standard was also the basis for the national 2010 “Formaldehyde Standards for Composite Wood Products Act” [26]. The newer standards have led to new UF resins that provided lower formaldehyde emissions [27], as well as opened the door for no-added formaldehyde (NAF) adhesives such as polymeric diphenylmethane diisocyanate resin, soy-based resins [28,29], and cross-linked poly(vinyl acetate) adhesives. The current accepted conditions for product testing formaldehyde emissions are what are considered typical conditions of 25 °C and 50% relative humidity. UF resins have been modified to meet these conditions, including CARB phase II-compliant and ultra-low-emitting formaldehyde (ULEF) UF resins, with the latter needing to provide lower emission levels. This progress has been made by most notably using melamine–formaldehyde fortification and copolymerization with melamine, lowering the formaldehyde-to-urea ratio and/or the addition of scavengers [30]. The question arises: Do these new products produce a cured adhesive that is more resistant to releasing formaldehyde than tradition UF adhesives?

Although both the newer UF and NAF resins are capable of passing the CARB phase II formaldehyde emissions limits, there is concern about the long-term emission potential of the cured UF adhesives, in particular when exposed to temperature or humidity conditions higher than specified in the current testing methodology for measuring formaldehyde emissions. The testing procedure for measuring formaldehyde conditions is set at “typical” interior conditions of 25 °C and 50% relative humidity. Unfortunately, homes and/or offices are not always maintained at these “typical” conditions; thus, wood products, and their human occupants, can be exposed to environments of higher temperature and higher humidity levels than specified in the standard testing procedure. It is these “non-typical” conditions that are the focus of this research project to determine if CARB phase II UF resin composite panels emit higher formaldehyde levels.

Even though less formaldehyde, but more formaldehyde capture agents, etc., are used in the newer UF resins, the question is whether the new urea–formaldehyde structure is unchanged in these adhesives, and whether they could remain susceptible to hydrolysis over the lifetime of the product. The sensitivity of cured UF adhesives to moisture has prevented them from being used in exterior applications [31], and it can cause bond failure in interior applications with high humidity [32]. Further details on the formaldehyde emission from composites were discussed in our prior publication [2]. 

In determining the UF contribution to formaldehyde emissions, it needs to be recognized that wood also produces formaldehyde, especially under conditions of composite manufacturing where temperatures exceed 100 °C [33,34]. However, this “native” formaldehyde has been shown to be transient and rapidly decreases to levels below those set by the standards [35,36]. 

**Figure 2:**
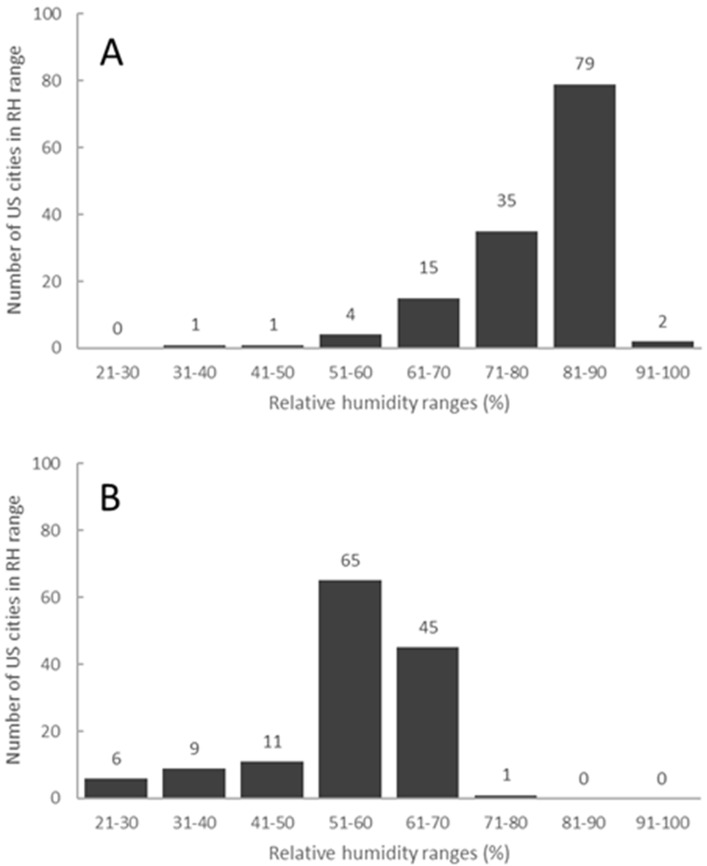
Relative humidity distribution of 137 US cities: (**A**) morning (average = 79%) and (**B**) evening (average = 55%).

The typical test conditions of 25 °C and 50% relative humidity assume air conditioning and good air flow in humid environments [37]. When a home air conditioning system is not operated continuously, or in the case of a trailer/recreational vehicle that may be subjected to extended periods of time at higher temperatures and/or higher humidity, typical conditions are exceeded. The data in Figure 2A (www.weather.com) and Figure 2B (www.cityrating.com/relativehumidity.asp) show that ambient conditions in the majority of the United States are in fact often much higher than 50% relative humidity in the morning and evening. The relative humidity is higher in the morning and it is known that the water absorption in wood has a hysteresis with the wood absorbing more water than it releases at the same temperature and humidity conditions [38]. Consequently, moisture picked up at night will not be readily lost during the day. Most notably, the southeastern region during the summer months is substantially higher in both relative humidity and temperature when compared with the test conditions. It is this finding that led us to evaluate composite panel emissions from new adhesives as a function of temperature and humidity.

The primary standard test method in the United States for measuring and regulating in North America formaldehyde emissions in composite wood panels is the ASTM E1333 large chamber test [39]. In E1333, samples are conditioned at 25 °C and 50% relative humidity (RH) for seven days and then tested at the same temperature and RH conditions. However, for non-standard conditions, the only report of the formaldehyde decay curves comparing CARB Phase II- and ULEF-compliant adhesives boards shows different decay curves compared with isocyanate boards [40]. Under the standard condition of 25 °C and 50% RH and the elevated condition of 29 °C and 75% RH, the MUF (melamine-amine formaldehyde) and phase II-compliant UF formaldehyde emissions did not decay as fast as the formaldehyde emissions of the isocyanate boards, and after 50 days, it never reached equally low emissions under the standard conditions compared to the isocyanate and ULEF boards. The work also showed that the elevated temperature and humidity increased the emissions of the MUF and UF boards by 2–3 times over “typical” conditions. 

In a previous paper [2], we showed that emissions from UF resin-bonded particleboard for the first 4 days had substantially increased when panels were exposed to elevated heat and humidity in the WKI (Fraunhofer Institute for Wood Research, Wilhelm-Klauditz-Institut WKI) bottle test compared to the standard conditions in the ASTM E1333 test procedure [2,34]. The utility of the WKI method for evaluating formaldehyde emissions under different conditions has been discussed by Roffael [16]. These small samples did not have their edges taped, but this is a less significant factor in a static test than in the dynamic flow tests that remove the formaldehyde as it is emitted. This study builds on our prior work by storing UF and NAF adhesively bonded particleboard samples exposed to 21 °C and 50% RH conditions from 0 to 186 weeks (3.5 years), followed by testing those samples at both typical 25 °C and 50% RH and elevated conditions (35 °C and 100% RH) in order to assess the panels’ long-term maximum potential for formaldehyde emissions when exposed to elevated conditions compared to “typical” conditions. Although the sensitivity of particleboards to heat and humidity has been reported previously for traditional types of UF resins, the information on the emissions of the newer CARB phase II-compliant UF boards after long-term exposure to “typical” conditions have not been examined.

## 2. Methods

**Samples**: Commercially produced 3/4-inch (19.0-mm) thick CARB phase II-compliant particleboard was obtained from a commercial collaborator. Samples were collected and shipped according to ASTM E1333, including wrapping in plastic until testing or exposure. In addition, samples of the same face and core furnish (Northwestern US pine species mix) were supplied to our laboratory where comparable NAF particleboard panels were made. Although the authors agree that a laboratory process is not an exact replication of commercial conditions, the laboratory NAF panels were processed under conditions as close to the commercial panels as possible using similar press temperatures, press times, board thicknesses, moisture contents, face-to-core furnish ratios, and adhesive concentrations. A pinch collar was used to match the greater internal steam pressures of commercial production. This steel collar was pressed into the panels around their edges to inhibit the release of steam, mimicking the conditions for a commercial size panel [41]. Table 1 shows that the commercial UF resin-bonded and laboratory soy NAF-bonded panels had similar physical properties of internal bond strength, modulus of rupture, and apparent density. 

The NAF adhesive used was based on Soyad™ adhesive technology (Solenis, Wilmington, DE, USA). Soyad technology uses a polyamidamine–epichlorohydrin cross-linking resin in combination with a soy dispersion made from soy flour and various diluents. The soy flour dispersions Soyad SD419 (face) and Soyad SD424 (core) were used. It should be noted that Soyad SD419 adhesive contains some urea to scavenge the native formaldehyde.

Both the commercially and laboratory produced panels were sanded, allowed to cool, and then wrapped and sealed in plastic within 48 h after hot-pressing and remained in plastic until initial testing or long-term exposure. The commercial particleboard samples were reported by the mill to be CARB phase II compliant (<0.09 ppm). Both types of products were unwrapped and cut into 0.3 m by 0.3 m samples that were stored in a room of 127 m^3^ that had normal lab traffic but was controlled to 21 ± 1.9 °C, 50 ± 3.3% RH.

**Figure 3:**
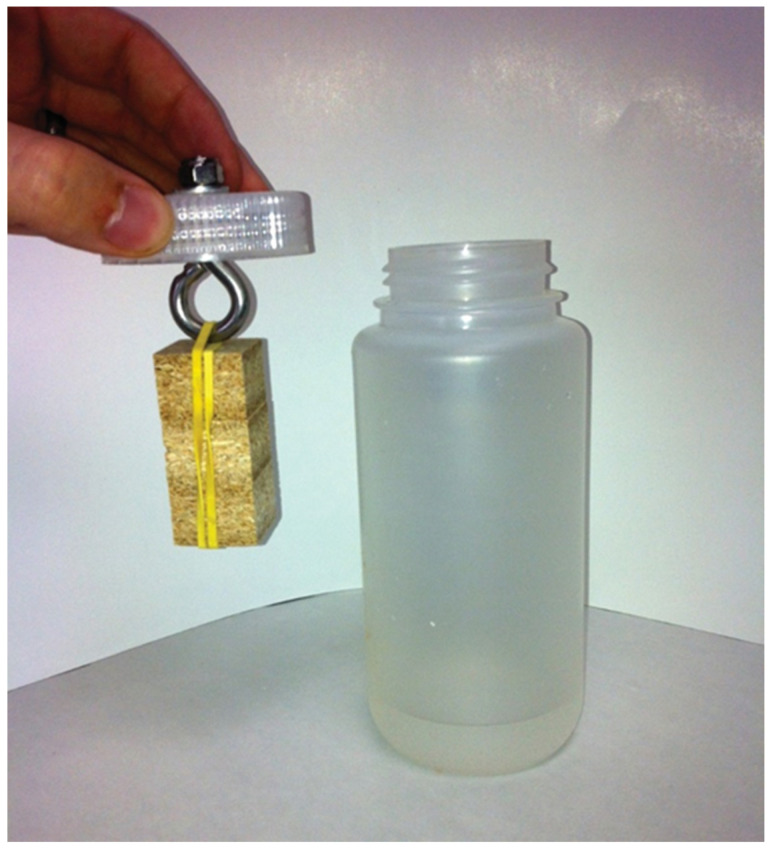
Sample setup for modified EN-717-3 method [44].

**Modified EN 717-3 (WKI Bottle Method) [42]**: A modified version of EN-717-3 was conducted, and Table 2 defines the specific modifications. Relative humidity was controlled as follows: 50% RH (with saturated Mg(NO_3_)_2_) and 100% RH (reverse osmosis H_2_O) [43]. Relative humidity values obtained via this method were confirmed to be within ±2% RH. Temperature was maintained using a water bath capable of holding temperatures within ±1 °C. The 25 °C and 50% RH are considered typical conditions, while the higher temperature and humidity are the same as prior testing conditions [2]. The moisture content of the specimens going into the emissions test was 8.8 ± 0.5% (dry wood basis). The test procedure illustrated in Figure 3 and the analysis method have been described in the literature in great detail [2]. Duplicate tests were performed for each condition and board type. The difference between replicates ranged from a low of 0.2% to a high of 59%, with an overall average of 13%. In general, the specimens with lower emissions had a higher variation between replicates. 

## 3. Results and Discussion

To study the basic science of formaldehyde emissions from different particleboards, a static formaldehyde emissions method was employed to assess the changes in emissions for composite wood products as a function of temperature and humidity, using a modified version of the EN 717-3 method, as outlined in Table 2. The modifications allowed the test to be run under different temperature and relative humidity conditions to better understand the formaldehyde emissions of composite panels under other conditions than the “typical” conditions as mandated by the current testing method E1333. The 100% RH is certainly much higher than typical, but accelerated tests are generally run under more severe conditions than normal exposures because of the shorter times in accelerated tests. Furthermore, it is not unreasonable to have non-air-conditioned homes in many parts of the country that approach this relative humidity condition.

Two particleboards were evaluated in this study with both boards made with the same furnish source with one commercially produced using a CARB phase II-compliant UF resin and the other laboratory produced (34 by 34-inch, 86.4 by 86.4-cm) with soy flour-based Soyad resin technology. Although careful attention was paid to reproduce the commercial process in a laboratory setting [41], the authors do recognize that this is a difference, but they do not consider the results or conclusions to be in question because of this difference. 

Particleboard samples of roughly 0.3 m by 0.3 m were kept in storage in an environmental conditioning room at 21 °C and 50% RH for various durations up to 186 total weeks (approximately 3.5 years). We will refer to this condition (21 °C and 50% RH) as standard temperature and humidity storage conditions. After storage at standard conditions for various periods of time, one each of UF and Soyad-resin bonded particleboard samples were removed and cut into 25.4 mm by 25.4 mm squares for testing. Special care was taken to keep these squares sealed in separate plastic bags between being cut up and testing to minimize any contamination or off-gassing before the modified WKI bottle tests. 

Two conditions were employed in the modified WKI bottle tests—typical temperature and relative humidity (25 °C and 50% RH) and elevated temperature and relative humidity (35 °C and 100% RH). The result was that UF and NAF resin-bonded particleboards, after being stored at standard conditions, were then tested for formaldehyde emissions at both typical and elevated conditions. The purpose of this test method was to determine the long-term formaldehyde emissions potential for both UF and NAF resin-bonded particleboard. We demonstrated in the previous paper in this series [2] that CARB phase II UF resin-bonded particleboard generated and emitted significantly greater amounts of formaldehyde when the panel was exposed to higher temperature and/or higher humidity. This study raised the question: Would the panel that was stored for 3.5 years exposed to the typical conditions still emit similarly greater amounts of formaldehyde when the panel was introduced to higher temperature and/or higher humidity, or was all of the volatile or labile formaldehyde already gone? The newer UF resins should have less formaldehyde polymers and less UF polymers with dimethyl ether segments that have been claimed as the sources of the formaldehyde emissions; plus, they also have formaldehyde capture additives. 

**Figure 4:**
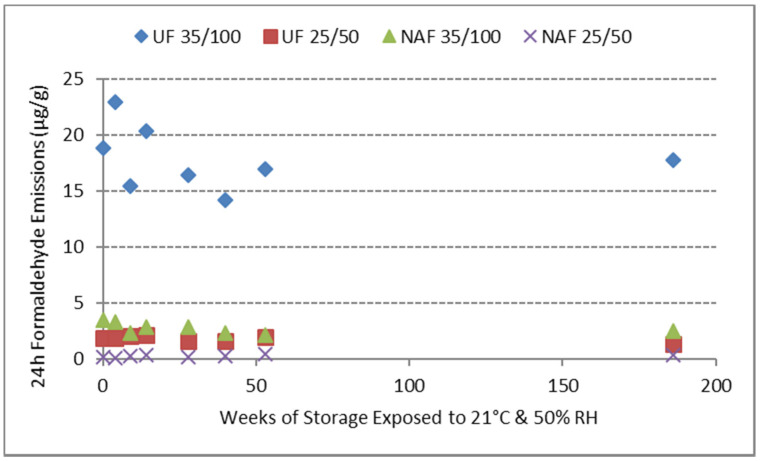
Formaldehyde emissions (ug/g dry wood) as a function of storage time.

Figure 4 shows the emissions over time for both the typical and elevated conditions. The prior study only involved 0 days of storage followed by WKI bottle tests that ranged from 24 to 96 h in duration [2], while this study involved WIKI bottle tests for matched samples stored exposed to standard conditions for 3.5 years. The horizontal axis represents the length of time samples were stored unwrapped at the standard storage condition (21 °C and 50% RH) before 24 h of exposure at either the typical (25 °C and 50% RH) or elevated (35 °C and 100% RH) test conditions. These results clearly show that the commercial UF resin-bonded panel still emitted significantly (about 7 times) greater formaldehyde levels when subjected to the elevated temperature and relative humidity condition, and that this potential for emissions at the elevated condition is maintained even after a long period of time (at least 3.5 years) at the standard storage conditions. 

Our previous paper looking at formaldehyde emissions from UF and Soyad resin-bonded particleboards at various temperature and relative humidity conditions attempted to assess the longer-term emissions by comparing 1-day WKI bottle tests of a fresh particleboard sample to a sample exposed to the typical conditions for 6 days before the 1-day test. That study showed about an 11% increase in formaldehyde emissions from the UF-bonded panel when the samples had previous exposure and about a 9% decrease in formaldehyde emissions from the Soyad adhesive-bonded panel when the samples had previous exposure. However, our conclusion was that this small change in absolute numbers was probably noise in the test, and there was no real reduction in formaldehyde emissions over time [2]. Figure 4 confirms that for particleboard made with either UF or NAF adhesives, long-term storage at standard conditions does not reduce formaldehyde emissions. 

The emission data are also shown in tabular form in Table 3. The effect of temperature and humidity on formaldehyde emissions seen in Figure 4 is similar to the effects seen with conventional UF resins by Myers and Nagaoka [5,11]. Although Myers and Nagaoka [11] did not conduct analyses at relative humidity levels above 75%, in a comprehensive literature survey, Myers was able to derive quantitative temperature and relative humidity factors at a wide range of temperature and relative humidity conditions [5]. Based on these equations, going from 30% to 90% RH at 25 °C is predicted to yield a 3-fold increase in formaldehyde emissions. This result is not outside the conclusion with CARB phase II-compliant particleboard of a 2–3 fold increase going from 25 °C, 50% RH to 35 °C, 75% RH [40]. An analysis of data in Table 3 shows that going from 25 °C and 50% RH to 35 °C and 100% RH yields an average 10-fold increase in emissions, although the small emission number for the typical conditions can lead to some over-representation of the magnitude of the increase. This 10-fold increase was regardless of resin type; however, the UF-bonded board, on average, had formaldehyde emissions 6.7 times greater than the NAF-bonded board at the same conditions. Although the numbers in this paper were measured using a static test rather than the dynamic test often used in the literature, the results agree reasonably well with prior studies and Myers’ prediction with conventional UF-bonded particleboard. The newer UF resins are able to meet the emissions requirements of the test method; however, they do not maintain low levels of formaldehyde emissions at elevated temperature and humidity, and furthermore, this potential for greater emissions is not diminished over time. 

## 4. Conclusions

The testing of formaldehyde emissions from CARB phase II-compliant particleboard panels bonded with a low-emitting urea formaldehyde (UF) resin and a no-added formaldehyde (NAF) soy resin were carried out at two different conditions after storage for up to 3.5 years, which were unwrapped to allow air exposure. Storage conditions were 21 ± 1.9 °C at 50 ± 3.3% RH, and the emissions testing conditions were typical (25 °C at 50% RH) and elevated (35 °C at 100% RH). After storage at standard conditions for up to 3.5 years, formaldehyde emissions remained relatively unchanged from day 1 emissions for both adhesive types and both test conditions. UF resin-bonded particleboard at the elevated conditions emitted much more formaldehyde than the NAF resin-bonded particleboard. This indicates that even CARB phase-II compliant particleboard made with a UF resin continues to be susceptible to the hydrolysis reaction that releases formaldehyde at higher than the typical conditions used in certification, and the board maintains this potential for elevated formaldehyde emissions well into its service life. Current UF resins are formulated to achieve the CARB phase II-compliant emission standards under the current typical test conditions of 25 °C at 50% RH. This paper, along with the previous paper in this series [2], demonstrates that these emission levels may be exceeded if building occupants do not insure that the temperature and humidity levels are continuously maintained near typical conditions. Thus, it is possible that current CARB phase II UF-adhesive bonded composites, even as much as 3.5 years into their service life, can still emit more formaldehyde than desired under certain indoor environments. With the commercial availability of NAF particleboards [45], one should carefully consider the use of NAF boards where the product is exposed to high humidity and temperatures.

## Figures and Tables

**Table 1:** Physical properties of commercial and laboratory panels. ^a^

	Internal Bond (psi) [MPa]	Modulus of Rupture (psi) [MPa]	Apparent Density (lb/ft^3^) [kg/m^3^]
Commercial UF-bonded	86.5 ± 9.0 [0.59 ± 0.06]	1999 ± 179 [13.78 ± 1.23]	44.6 ± 1.3 [714 ± 21]
Laboratory NAF-bonded	91.2 ± 10.1 [0.63 ± 0.07]	1973 ± 111 [13.60 ± 0.77]	46.8 ± 1.1 [750 ± 18]

^a^ UF = urea formaldehyde; NAF = no added formaldehyde.

**Table 2:** Summary of modifications to EN-717-3.

	EN-717-3	Our Method
Temperature	40 °C	25 °C and 35 °C
Test duration	3 h	24 h
Relative humidity	100%	50% and 100%

**Table 3:** Formaldehyde emissions (ug/g dry wood) as a function of storage time.

Weeks of Storage at 21 °C and 50% RH	UF		NAF	
	25 °C and	35 °C and	25 °C and	35 °C and
	50% RH	100% RH	50% RH	100% RH
0	1.9	19	0.19	3.5
4	1.8	23	0.10	3.3
9	2.0	15	0.28	2.4
14	2.2	20	0.34	2.9
28	1.6	16	0.18	2.8
40	1.6	14	0.25	2.3
53	2.0	17	0.47	2.1
186	1.4	18	0.38	2.5
Overall Average	1.8	18	0.27	2.7
X-increase	10		10	
